# Seizures and epilepsy in MOG antibody-associated disease: from cortical neuroimmunology to clinical practice

**DOI:** 10.3389/fimmu.2026.1966487

**Published:** 2026-10-06

**Authors:** Chun Li, Liangliang Hao, Xueyan Liu, Ji Zhou, Weining Ma

**Affiliations:** 1 Department of Pediatrics, Shengjing hospital of China Medical University, Shenyang, China; 2 Department of Pediatrics, Shenyang Children’s Hospital, Shenyang, China; 3 Department of Neurology, Beijing Children's Hospital, Capital Medical University, National Center for Children's Health, Beijing, China; 4 Department of Neurosurgery, Shengjing Hospital of China Medical University, Shenyang, China

**Keywords:** blood-brain barrier, cerebral cortical encephalitis, epilepsy, epileptogenesis, flames, interleukin-6, MOGAD, myelin oligodendrocyte glycoprotein

## Abstract

Myelin oligodendrocyte glycoprotein antibody-associated disease (MOGAD) is an inflammatory demyelinating disorder distinct from multiple sclerosis (MS) and aquaporin-4-IgG-positive neuromyelitis optica spectrum disorder (AQP4-NMOSD). Seizures are an important but incompletely characterized manifestation. Their reported prevalence is strongly age-dependent—approximately 30% in pediatric-onset versus 7% in adult-onset cohorts, although these estimates are influenced by referral and publication bias—and clusters around cerebral cortical encephalitis (CCE) and FLAIR-hyperintense lesions in anti-MOG-associated encephalitis with seizures (FLAMES). This raises a mechanistic question: why is cortical MOGAD particularly ictogenic, and how does demyelination interact with lesion anatomy and inflammatory composition to promote cortical hyperexcitability? This review places the neuroimmunology of MOGAD-related seizures at its center. We summarize the epidemiology, age gradient and clinical spectrum—CCE/FLAMES, acute disseminated encephalomyelitis (ADEM)-associated seizures, isolated sentinel seizures, status epilepticus, new-onset refractory status epilepticus (NORSE), and MOG–N-methyl-D-aspartate receptor (NMDAR) overlap—together with the distinction between acute symptomatic seizures and autoimmune-associated epilepsy. We then examine MOG-IgG effector biology, the CD4+ T-cell- and macrophage-predominant pathology, B-cell compartmentalization, the meningo-cortical axis, and the Th17/interleukin-6-weighted cerebrospinal fluid (CSF) milieu. Candidate downstream pathways of inflammatory ictogenesis are presented with an explicit separation between MOGAD-specific associations and mechanisms extrapolated from epilepsy. Finally, we translate this framework into differential diagnosis, assay interpretation, electroencephalographic evaluation, immunotherapy-first management, antiseizure medication (ASM) review, neuropsychological follow-up and testable research priorities.

## 1 Introduction

Myelin oligodendrocyte glycoprotein (MOG) is a quantitatively minor but immunologically dominant constituent of central nervous system (CNS) myelin. Its expression is largely restricted to the CNS, although low-level peripheral nervous system expression has been reported (1, 2). MOG occupies the outermost myelin lamellae and oligodendrocyte plasma membrane, and its extracellular immunoglobulin-like domain is accessible to circulating autoantibodies (1). Conformation-sensitive cell-based assays (CBAs) using full-length human MOG transformed a historically unreliable marker into the defining biomarker of MOGAD (1, 3).

The 2023 International MOGAD Panel proposed criteria formalized this entity, requiring a core clinical demyelinating attack, a positive MOG-IgG test with titer-dependent supporting features, and exclusion of better-fitting alternative diagnoses (3). Critically, the panel listed cerebral cortical encephalitis (CCE) with or without seizures among the six core clinical attacks, thereby recognizing that cortical, seizure-predominant presentations belong to the disease rather than representing coincidental comorbidity (3–5).

Seizures occupy a distinctive position within this spectrum. They are comparatively uncommon in MS and rare in aquaporin-4-IgG-positive neuromyelitis optica spectrum disorder (AQP4-NMOSD), but occur in a substantial fraction of patients with MOGAD. A synthesis of 178 studies reported seizure prevalence of 30.6% (95% CI 28.0–33.3) in pediatric-onset and 7.0% (95% CI 4.1–11.2) in adult-onset cohorts (6, 7); these figures require cautious interpretation because the evidence base mixes clinical cohorts with case reports and small series. In single-center cohorts, seizures attributable to CNS autoimmunity occur in roughly 10–16% overall, again with pediatric predominance (8). The phenotype most tightly associated with seizures is CCE, first described in MOG-antibody-positive adults and subsequently characterized as FLAMES (9, 10).

This clinical picture generates the central question of this review. MOG is expressed on oligodendrocytes and myelin rather than neurons, and demyelination can itself alter axonal conduction and excitability. Demyelination alone, however, is unlikely to account fully for the high seizure susceptibility of cortical MOGAD. The more specific question is how myelin injury interacts with pial and cortical lesion location, cellular inflammation, barrier dysfunction and age-dependent network properties. A clinically inseparable question is whether seizures are acute symptomatic events or, in a subgroup, mark an enduring predisposition—autoimmune-associated epilepsy (11).

Our thesis is that ictogenesis reflects an interaction among demyelination, lesion anatomy and inflammatory composition. When inflammation is centered at the pial surface and superficial cortex, cytokine, glial, complement and vascular disturbances can impinge directly on established pathways of ictogenesis (12, 13). This formulation does not treat demyelination and inflammation as competing explanations; it asks why a particular anatomical and immunological form of demyelinating inflammation is unusually seizure-prone, and why it resolves in most patients but consolidates into epilepsy in a minority.

Earlier reviews have defined the MOGAD meningo-cortical spectrum, compared seizures across inflammatory demyelinating diseases and systematically characterized MOGAD-related epilepsy (6, 7, 14). The added value of the present review is an evidence-audited mechanistic synthesis linked to practical diagnostic and treatment decisions. It generates two testable predictions: at comparable inflammatory burden, cortical or leptomeningeal lesions should confer greater seizure risk than noncortical lesions; and persistent barrier dysfunction or a sustained inflammatory CSF signature after attack resolution should distinguish patients who develop chronic epilepsy from those with only acute symptomatic seizures.

## 2 Methods

This article is a narrative review based on expert selection of sources identified in PubMed/MEDLINE and Web of Science from database inception through September 1, 2026. Search terms combined “MOG”, “myelin oligodendrocyte glycoprotein”, “MOGAD”, “cortical encephalitis”, “FLAMES”, “seizure”, “status epilepticus”, “epilepsy”, “epileptogenesis”, “neuroinflammation”, “blood-brain barrier”, “astrocyte”, “microglia”, “complement”, “interleukin-1”, “interleukin-6” and “tumor necrosis factor”. Reference lists of relevant articles and major reviews were also examined. Cohorts, systematic reviews, neuropathological series, consensus statements and primary mechanistic studies were prioritized; case reports were retained when they documented otherwise unrepresented phenomena such as NORSE. We did not use a prespecified protocol, dual independent screening, a selection flow diagram or formal risk-of-bias grading; the review should therefore not be interpreted as a systematic review.

## 3 Epidemiology and the age gradient

### 3.1 Prevalence across the lifespan

MOGAD is uncommon, but incidence and prevalence vary across geography, age structure, assay availability and case ascertainment. A global epidemiological review found substantial regional heterogeneity and emphasized that direct comparisons remain limited by differing definitions and referral pathways (15). More recent population-based estimates from the United States, Martinique and Denmark provide useful regional data, including incidence in the range of 3.4–4.9 per million per year and prevalence around 20–40 per million in some settings, but should not be treated as universal global ranges (15–17).

The most comprehensive synthesis included 2,487 patients from clinical cohorts and a further 337 patients from case reports and small series, and reported seizure prevalence of 30.6% in pediatric-onset and 7.0% in adult-onset disease (6). This striking gradient is directionally consistent with hospital cohorts, but it is not an unbiased global prevalence estimate: referral-center enrichment and the disproportionate publication of dramatic encephalitic or seizure presentations—especially in children—can inflate the pediatric estimate. It should therefore be interpreted alongside population-based and consecutive cohorts. Among 213 patients meeting MOGAD criteria at one tertiary center, seizures attributable to CNS autoimmunity occurred in 23 (10%), 19 of whom had pediatric-onset disease (8). In a consecutive cohort of 135 patients without pre-existing epilepsy or concurrent NMDAR antibodies, 19 developed seizures (18). Among 235 children with MOG antibodies, 33 met criteria for non-ADEM encephalitis and seizures were present in 73% of that selected subgroup (19).

Comparative studies confirm that this frequency is disease-specific rather than a generic feature of CNS demyelination. Acute symptomatic seizures occur more frequently in MOGAD than in MS or AQP4-NMOSD, and in AQP4-NMOSD in particular seizures remain distinctly rare (7, 20, 21).

### 3.2 Age as an immunological variable

The two- to fourfold excess of seizures in pediatric-onset MOGAD is not merely a reflection of the higher frequency of ADEM in children. Pediatric-onset patients are significantly more likely to experience status epilepticus and encephalopathy, whereas adult-onset patients more frequently show CSF pleocytosis; adults more often have parietal lobe involvement and fewer temporal lobe lesions; and, most importantly for prognosis, chronic epilepsy is significantly more prevalent among pediatric-onset patients despite comparable use of immunotherapy and antiseizure medication (6). Pediatric-onset patients in that synthesis were also significantly more likely to be female (6).

Age plausibly modifies both seizure susceptibility and the likelihood of persistent epilepsy. The immature cortex has different chloride homeostasis, excitatory–inhibitory balance, synaptic plasticity and seizure propagation, while the developing neurovascular unit, astrocytes and microglia respond differently to inflammatory injury (22–24). Pediatric MOGAD also more often assumes an ADEM or encephalitic phenotype, increasing the probability that inflammation reaches cortex. A testable developmental model is therefore a two-hit interaction: an age-dependent excitable network and glial–vascular response are superimposed on a higher frequency of cortex-directed inflammation. Prospective pediatric–adult studies should test whether age modifies the effect of lesion location or CSF inflammation rather than treating age as a simple covariate.

## 4 The clinical phenotypic spectrum of MOGAD-related seizures

Seizures in MOGAD have a recognizable profile: they occur more often than in other inflammatory demyelinating diseases, usually accompany an acute attack, cluster with CCE and ADEM, and remit in most patients (6–8). **Table 1** summarizes the principal phenotypes.

**Table 1:** Clinical phenotypes of MOGAD-related seizures.

Phenotype	Typical age	Seizure characteristics	Key imaging/CSF features	Outcome
Cerebral cortical encephalitis/FLAMES	Children and adults; FLAMES described in both	Focal, often focal-to-bilateral tonic-clonic; near-universal (85–100%)	Cortical FLAIR hyperintensity ± swelling, unilateral (FLAMES) or multifocal; sulcal/leptomeningeal enhancement; CSF pleocytosis, raised opening pressure in children	Corticosteroid-responsive; median time to seizure control ~1.25 months; MRI resolves; relapse in up to 50% (25, 26)
ADEM-associated	Younger children (median ~5.7 y)	Seizures in the context of encephalopathy and multifocal deficits	Multifocal white and deep grey matter lesions	Generally good; multiple ADEM-like relapses predict epilepsy (7, 19)
Isolated/sentinel seizures	Predominantly children	Clustered focal motor seizures; may precede demyelinating syndrome	Initial MRI normal in up to 24%; new lesions on repeat imaging in ~50% rescanned	Diagnosis frequently delayed; MOG-IgG testing warranted (19, 27)
Status epilepticus/NORSE/super-refractory SE	Predominantly pediatric-onset	Convulsive or focal SE; may be refractory to anesthetic agents	Extensive uni- or bilateral cortical involvement	Rare but severe; rationale for early anti-IL-6 therapy (6, 28, 29)
MOG–NMDAR overlap (MNOS)	Any	Seizures with prominent psychiatric, movement and autonomic features	Cortical and/or ADEM-like changes; dual antibody positivity	Antibody co-positivity in 37.3% of MOGAD epilepsy patients, 88.3% of these NMDAR (6, 8, 30)

The practical consequence is conditional rather than universal. In a compatible inflammatory demyelinating or encephalitic syndrome, seizures can strengthen suspicion of MOGAD. Recurrent seizures outside an acute attack should prompt evaluation for autoimmune-associated epilepsy, MOGAD relapse, structural sequelae and alternative etiologies; they should not automatically be dismissed as unrelated to MOGAD.

### 4.1 Cerebral cortical encephalitis and FLAMES

CCE is the phenotype in which seizures are most consistently and most prominently expressed, and in patients with MOGAD-related epilepsy it is the most common presentation in both age groups (6). Its clinical core is a subacute syndrome of seizures, headache and fever, frequently with encephalopathy and focal deficits, accompanied by cortical FLAIR hyperintensity with swelling, variable leptomeningeal and sulcal gadolinium enhancement, and CSF pleocytosis (4, 14).

FLAMES designates the radiologically distinctive unilateral form (9, 10). It provides a particularly informative model, because in FLAMES cortical inflammation is essentially the only pathology present: there is no confounding white matter demyelination, no optic neuritis and no myelitis, and yet seizures are near-universal. In a systematic review of 39 adult cases of MOGAD CCE, seizures were present in 85%, headache in 82%, cortical symptoms in 64%, fever in 64% and altered consciousness in 54%; all cases showed cortical FLAIR abnormality; EEG was abnormal in 76% of those tested; CSF white cell count was elevated in 90% and protein in 67%; and CSF cytology showed monocytic predominance in 69% of those with cytological data (31). Notably, in the 15 cases in which MOG-IgG was assayed in paired samples, 14 (93%) showed a higher titer in serum than in CSF, indicating that CCE is not typically driven by an isolated intrathecal antibody response (31). Similar profiles have been reported in other adult case series and literature reviews (32, 33).

In a UK national series of eight adults with MOGAD CCE, all had seizures, five of eight showed cerebral swelling, five of seven had CSF pleocytosis, the median time to seizure control was 1.25 months, and all clinical features and MRI abnormalities eventually resolved — although four of eight (50%) subsequently relapsed, at a median of 6.4 months (25). The pediatric picture is comparable but with even higher seizure penetrance: among 123 children with MOGAD at a single center, nine met criteria for FLAMES; all nine had seizures (six focal), three had status epilepticus, seven had raised CSF opening pressure (240–280 mmH_2_O), interictal epileptiform discharges were present in six of seven EEGs, unilateral cortical FLAIR hyperintensity was present in all nine (frontal in eight), and sulcal or meningeal enhancement was seen in five (26). All nine achieved a good outcome and remained relapse-free (26). Raised intracranial pressure and headache are increasingly recognized as accompanying features of pediatric MOGAD encephalitis and may themselves reflect the meningeal component of the process (27, 34).

### 4.2 Peri-ictal MRI change, EEG and differential diagnosis

Reverse causality must be considered because prolonged or clustered seizures can themselves cause transient cortical FLAIR hyperintensity, swelling, diffusion abnormality, hyperperfusion and occasional leptomeningeal enhancement (35). Features favoring primary MOGAD inflammation include inflammatory symptoms or imaging change before seizure onset, CSF pleocytosis, a compatible MOGAD core phenotype, lesion evolution beyond the expected peri-ictal period and a coordinated clinical–radiological response to immunotherapy. Conversely, close electroclinical concordance, prominent diffusion or perfusion change immediately after status epilepticus and rapid resolution without an inflammatory CSF profile support a peri-ictal contribution. The two processes may coexist, and serial MRI is often more informative than a single scan.

Electroencephalography (EEG) commonly shows focal or regional slowing, lesion-concordant interictal epileptiform discharges or focal ictal patterns; a normal routine EEG does not exclude CCE. Continuous EEG is appropriate for persistent altered consciousness, suspected nonconvulsive seizures or status epilepticus. Important mimics include herpes simplex encephalitis (temporal/insular predilection, hemorrhage or marked diffusion restriction and virological evidence), mitochondrial encephalopathy with lactic acidosis and stroke-like episodes (MELAS; nonvascular stroke-like lesions and metabolic or genetic clues), Rasmussen encephalitis (progressive unilateral deficits, epilepsia partialis continua and hemispheric atrophy), neuronal-surface antibody encephalitis (psychiatric, movement, autonomic or syndrome-specific antibody features) and isolated peri-ictal change. MOG-IgG testing should be targeted to a compatible inflammatory phenotype rather than ordered indiscriminately for all new-onset seizures.

### 4.3 ADEM and encephalopathy-associated seizures

In children, ADEM is the other major context in which seizures occur. In the tertiary-center seizure cohort, 8 of 23 patients (35%) had CCE alone, 6 (26%) had ADEM alone, 7 (30%) had both, and the remaining 2 (9%) had other MOGAD encephalitic presentations (8). Multiple relapses with ADEM-like phenotypes have been associated with subsequent epilepsy in children (7). Children with non-ADEM encephalitis were older than those with ADEM, more often required intensive care, received corticosteroids later and were more likely to carry an epilepsy diagnosis at follow-up (6/33 vs 1/74; p = 0.003) (19).

### 4.4 Isolated and sentinel seizures

A clinically important minority present with seizures before a recognizable demyelinating syndrome. Initial brain MRI was normal in 8 of 33 children with MOG-antibody encephalitis, and repeat imaging disclosed new lesions in 7 of 13 rescanned (19). A normal initial MRI therefore does not exclude MOGAD when the overall syndrome is inflammatory and compatible; repeat imaging and targeted MOG-IgG testing may be warranted (19). Testing unselected seizure populations is inappropriate. Serum live CBA is preferred when available; low-positive results, especially from fixed assays, require a compatible core phenotype and supporting clinical or radiological features because positive predictive value falls markedly at low pretest probability and low titers (3, 36, 37). CSF testing may be informative in selected high-suspicion, serum-negative cases, but apparently CSF-restricted positivity has yielded different predictive values in enriched and unselected referral cohorts and demands careful exclusion of mimics (38, 39).

### 4.5 Status epilepticus, NORSE and the severe end of the spectrum

Status epilepticus is more frequent in children than adults: it occurred in 13% of patients with seizures in one cohort (8) and in three of nine children with FLAMES in another (26). MOGAD is a rare cause of new-onset refractory status epilepticus (NORSE), defined as refractory status epilepticus in a patient without active epilepsy or another clear acute structural, toxic or metabolic cause (28, 29). These cases show that an ordinarily self-limited, steroid-responsive process can become catastrophic and require rapid parallel antiseizure, diagnostic and immunotherapeutic escalation.

### 4.6 MOG–NMDAR overlap syndrome

Coexisting antibodies are common. In the systematic synthesis of MOGAD-related epilepsy, co-positivity with antibodies associated with other encephalitides was present in 37.3% of patients, of which anti-NMDA receptor IgG accounted for 88.3%, without age-dependent differences (6). In hospital cohorts, NMDAR-IgG coexistence is reported in around 13% of patients with MOGAD and seizures (8). Adults with dual NMDAR and MOG positivity display a phenotype that borrows from both diseases (30). The immunological basis of this association is unresolved; proposed explanations include a shared upstream trigger such as a preceding infection, BBB disruption in one disease exposing antigens of the other, and epitope spreading following tissue injury. Whatever the mechanism, dual positivity is clinically consequential, since NMDAR antibodies have well-defined direct effects on synaptic function (see Section 6) and the two antibodies may act additively on cortical excitability.

### 4.7 Acute symptomatic seizures versus autoimmune-associated epilepsy

The distinction drawn by the International League Against Epilepsy between acute symptomatic seizures secondary to autoimmune encephalitis and autoimmune-associated epilepsy, an enduring predisposition that persists beyond the acute inflammatory phase, is the central clinical question in MOGAD (11). In MOGAD the answer is reasonably clear: most seizures are acute symptomatic events.

Of 23 patients with MOGAD and seizures followed at a tertiary center, 21 (91%) were seizure-free at last follow-up. Three (13%) had developed drug-resistant epilepsy, but one of those three later became seizure-free after epilepsy surgery; the categories therefore overlap rather than sum to 24 patients (8). Although 60% relapsed, only 5 of 23 had further encephalitic episodes with seizures (8). In a separate cohort followed for a median of 92 months, 7 of 19 patients with seizures had at least one recurrence during MOGAD remission and 5 remained on ASM at last follow-up (18). Chronic epilepsy was more common after pediatric-onset disease (6).

Two points follow that are easy to conflate. Seizure recurrence in MOGAD is not simply a marker of disease relapse: in the cohorts above, relapse was common while recurrent seizures were not, and the recurrences that did occur happened predominantly during clinical remission. A small group therefore has genuine autoimmune-associated epilepsy rather than repeated acute symptomatic events. That group is enriched among children and among patients with severe or repeated encephalitic attacks, and it is the group for whom continued antiseizure medication is justified.

## 5 Immunopathogenesis: from MOG autoimmunity to cortical injury

### 5.1 MOG as an autoantigen

MOG is a type I transmembrane glycoprotein of the immunoglobulin superfamily, encoded within the major histocompatibility complex region and expressed largely, but not absolutely, within the CNS (2, 40). Its extracellular IgV-like domain contains conformation-dependent epitopes recognized by pathogenic antibodies (41). Its surface location makes it accessible after barrier disruption, while epitope conformation explains why full-length live or validated fixed CBAs—not denaturing assays—are required for diagnosis (1, 37, 42).

### 5.2 The effector repertoire of MOG-IgG

MOG-IgG in MOGAD is predominantly of the complement-activating IgG1 subclass (1, 42). Several effector mechanisms have been demonstrated experimentally and are not mutually exclusive:

Complement-dependent cytotoxicity (CDC). Human MOG antibodies bound to surface MOG activate the classical pathway and produce complement-dependent killing of MOG-expressing cells; the degree of CDC correlates with antibody titer and epitope specificity (43, 44). In cell and slice systems, MOG-IgG in the presence of complement produces oligodendrocytopathy — a pattern distinct from the astrocytopathy induced by AQP4-IgG (45).

Antibody-dependent cellular cytotoxicity and Fc-mediated effector recruitment. MOG-IgG engages Fcγ receptors on natural killer cells, macrophages and microglia, providing a route to tissue injury that does not require complement and that recruits the innate compartment directly into the lesion (42).

Antigen modulation and cytoskeletal disruption. Antibody binding can induce redistribution and internalization of surface MOG with secondary disruption of the oligodendrocyte cytoskeleton and microtubule organization, causing functional myelin compromise without frank lysis (42).

Facilitation of T-cell-mediated injury. Patient-derived monoclonal MOG antibodies are pathogenic *in vivo* but characteristically require a permissive T-cell-mediated inflammatory environment to gain CNS access and produce demyelination (46). This is a crucial qualification: in MOGAD, the antibody is best regarded as an amplifier acting within a T-cell-initiated lesion rather than as a self-sufficient effector.

### 5.3 The T-cell arm and immunogenetic context

The CD4+ dominance of MOGAD lesions makes T cells more than a permissive background. Human data remain limited: a small study of 24 patients detected subtle CD4+ responses to selected MOG peptide regions, including granulocyte–macrophage colony-stimulating factor responses, but did not establish a disease-defining clonotype or a cortex-specific repertoire (47). CSF cytokine profiles are compatible with Th17-related inflammation, yet cytokines cannot identify the antigen specificity or causal role of infiltrating T cells. Unlike several autoimmune diseases, a Dutch cohort found no strong HLA class I or II association with MOGAD, emphasizing immunogenetic heterogeneity rather than a single dominant restriction element (48). Direct comparisons of blood, CSF and lesion T-cell receptor repertoires across CCE, ADEM and optic neuritis are a major unmet need.

### 5.4 Neuropathology: perivenous, subpial, CD4-dominant

The single most informative neuropathological dataset in MOGAD is the immunopathological analysis of biopsy tissue from 11 patients, two of whom had cortical encephalitis (49). Its findings shape any mechanistic account:

- The dominant lesion architecture was ADEM-like perivenous demyelination (153 of 167 lesions), with a “fusion” pattern concentrated at the cortico-medullary junction and in white matter (11 of 167), and only three confluent plaques across two cases — a distribution quite unlike the confluent plaques of MS (49).
- Sixty of 167 lesions, predominantly in early disease, showed MOG-dominant myelin loss with relatively preserved oligodendrocytes, distinguishing MOGAD from the myelin-associated-glycoprotein-dominant oligodendrogliopathy of AQP4-NMOSD (49).
- The infiltrate was CD4^+^ T-cell-dominant (CD4^+^ 1281 ± 1196 vs CD8^+^ 851 ± 762 cells/mm², p < 0.01) with abundant CD68^+^ macrophages (1814 ± 1188 cells/mm²) and a B-cell component (CD20^+^ 468 ± 817 cells/mm²) — again a departure from the CD8^+^-predominant pattern of MS (49).
- Humoral effector footprints—perivascular activated complement and immunoglobulin—were only occasional and far less frequent than in AQP4-NMOSD (49). This creates a complement paradox: MOG-IgG is usually complement-fixing IgG1 and mediates complement-dependent cytotoxicity *in vitro* (43–45), yet tissue deposition is sparse and anatomically inconsistent. Timing of biopsy, lesion heterogeneity, Fc-receptor-mediated phagocytosis and complement-independent antigen modulation may explain part of the discrepancy; no study has demonstrated complement-dependent synaptic tagging in MOGAD cortex.
- Critically for the present argument, subpial lesions with perivenous demyelination were observed in both ADEM and cortical encephalitis (49).

Two points matter for seizures. The humoral response is demonstrable but is not the dominant effector arm in tissue; cellular immunity, and CD4+ T cells in particular, are central (50, 51). More importantly, MOGAD lesions preferentially involve the subpial cortex and the cortico-medullary junction, that is, the compartments immediately adjacent to cortical grey matter. Subpial demyelination associated with meningeal inflammation is a well-documented motif in multiple sclerosis cortical pathology, and its presence in MOGAD supplies the anatomical link between an anti-myelin response and cortical dysfunction.

### 5.5 The meningo-cortical axis

Bringing together imaging, CSF and pathology, MOGAD CCE is best conceptualized not as a white matter disease that happens to reach the cortex but as a meningo-cortical process (14). The supporting observations are: frequent leptomeningeal and sulcal gadolinium enhancement, present in 65% of patients with seizures in one cohort (8) and in 5 of 9 children with FLAMES in another (26); consistent CSF pleocytosis with monocytic and lymphocytic predominance (31); raised CSF opening pressure (26, 34); and subpial pathology on biopsy (49).

The mechanistic significance of this localization is plausible but inferential. Leptomeningeal inflammation and subpial pathology place soluble mediators near superficial cortex (14, 49). Because layers I–III contain dense synapses, pyramidal-cell apical dendrites and horizontal connections supporting intracortical synchronization, a pial-to-cortical gradient could preferentially disturb excitable networks. Such a concentration gradient has not been measured in MOGAD and should be tested with spatial pathology, imaging or compartment-resolved CSF sampling. This anatomical hypothesis contrasts with deeper noncortical lesions that more often produce conduction failure and focal deficits (7) (**Figure 1**).

**Figure 1:**
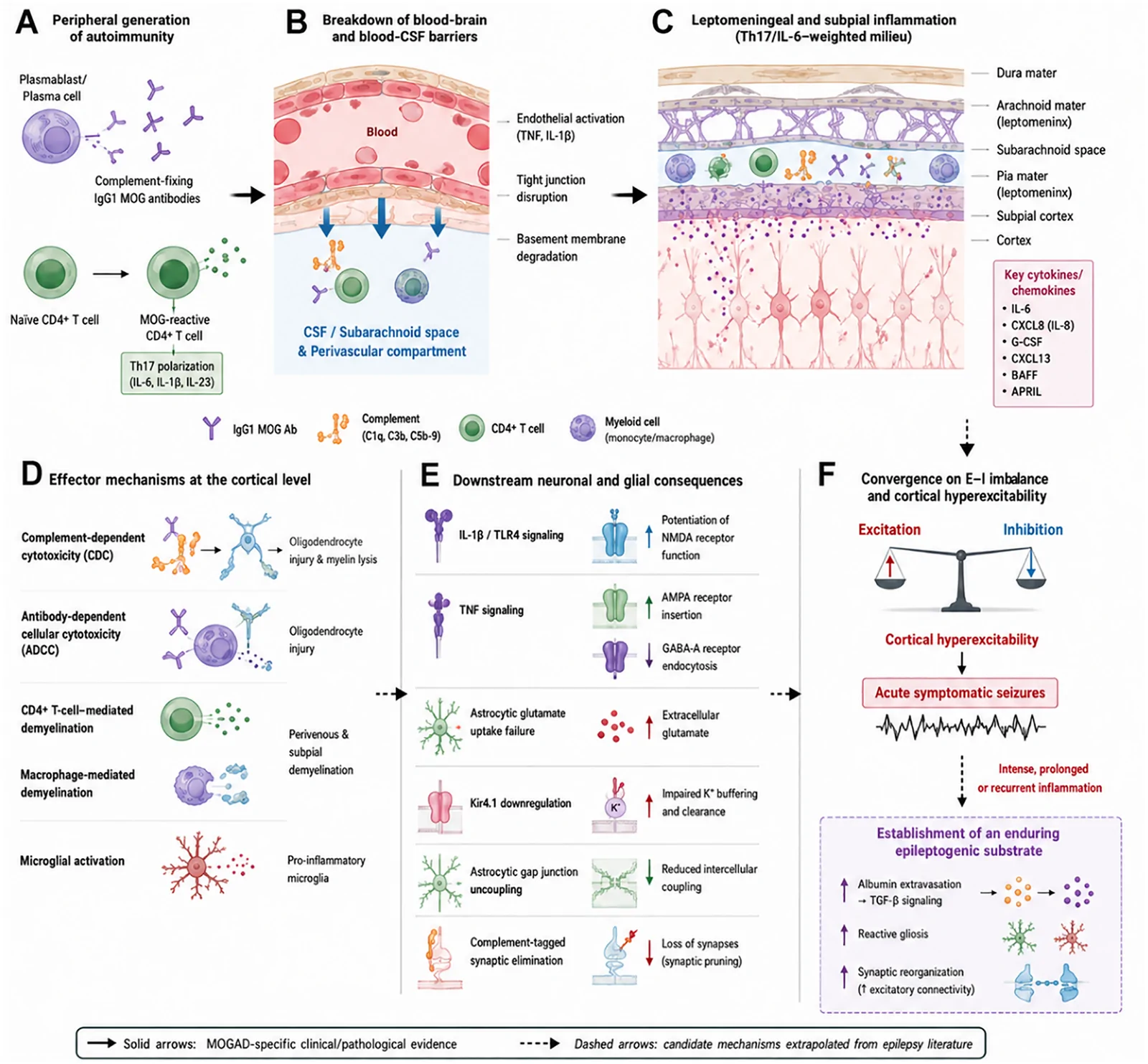
The meningo-cortical axis in MOGAD-related seizures. Solid connectors denote steps supported by MOGAD clinical, CSF or pathological observations; dashed connectors denote proposed transitions inferred from epilepsy biology. The corrected meningeal anatomy distinguishes dura, arachnoid and pia, with the subpial region located within superficial cortex rather than treated as a space. **(A–D)** summarize MOGAD-specific context; **(E, F)** show candidate downstream ictogenic pathways. The figure was created and revised using OpenAI image-generation tools and assembled by the authors. All schematic elements are original and no third-party copyrighted material was reproduced.

### 5.6 The CSF inflammatory milieu: a Th17/IL-6-weighted signature

The CSF cytokine profile in MOGAD has been characterized in both adults and children and is directly relevant to the mechanistic argument. In 29 patients with MOG-IgG-positive disease sampled in the acute phase before treatment, CSF levels of interleukin (IL)-6, IL-8/CXCL8, G-CSF, GM-CSF, interferon-γ, IL-10, IL-1 receptor antagonist, MCP-1 and MIP-1α were significantly elevated relative to MS, with no cytokine differing significantly from AQP4-NMOSD — a coordinated Th17-weighted signature with Th1 and regulatory components (52). Comparable B-cell-, Th17- and neutrophil-related cytokine and chemokine elevations have been reported in pediatric MOG antibody-associated demyelination (53). Elevated CSF IL-6 is likewise a hallmark of AQP4-NMOSD, where it correlates with disease activity and provided the rationale for IL-6 receptor blockade (54).

A recent study of 50 children with MOGAD reported higher CSF IL-6 and CXCL8 in a combined encephalitic group (ADEM plus CCE) than in optic neuritis, together with broader Th17-, B-cell- and neutrophil-related mediator elevations (55). The sample was small, ADEM and CCE were grouped despite biological heterogeneity, and the report did not clearly establish correction across all multiple cytokine comparisons. The finding should therefore be treated as phenotype association rather than mechanism. Only the CXCL13 CSF-to-serum ratio correlated with the ΔMOG-IgG ratio, a result compatible with—but not proof of—greater leptomeningeal B-cell support in encephalitic presentations (55).

This is one of the few observations linking an immune variable to a seizure-prone MOGAD phenotype, but it remains correlational. IL-6 and CXCL8 have plausible barrier and excitability effects in other systems, yet neither mediator has been shown to cause seizures in MOGAD. The result supports prospective biomarker and treatment-response studies; it does not by itself establish IL-6-directed therapy as an antiseizure mechanism.

### 5.7 B-cell compartmentalization, CSF-restricted antibody and epitope specificity

In paired samples from adult CCE, serum MOG-IgG titers exceeded CSF titers in 14 of 15 cases, favoring predominantly peripheral antibody production followed by barrier-dependent entry rather than autonomous intrathecal synthesis (31). CXCL13, BAFF and APRIL elevations may support local B-cell recruitment or survival, but they do not establish that the pathogenic antibody is produced within CSF. Reports of serum-negative, CSF-positive MOG-IgG complicate this model: CSF testing identified additional cases in a highly selected compatible cohort, whereas CSF-restricted positivity had low predictive value in an unselected national referral setting (38, 39). Such results require a compelling phenotype, appropriate assay controls and exclusion of alternative diagnoses.

Epitope specificity may also shape phenotype. In a large adult cohort, reactivity to the dominant P42 epitope was common, while non-P42 reactivity was associated with relapse risk (56). No validated epitope signature yet predicts cortical encephalitis rather than optic nerve or spinal disease. Linking epitope, Fc function, compartmental antibody index and lesion location in prospective cohorts is therefore more informative than treating serum titer alone as a mechanistic proxy.

### 5.8 The mechanistic gap: an honest appraisal

It is important to state plainly what has not been demonstrated. There is no direct evidence that MOG-IgG causes seizures, and no neuronal antigen has been identified for it. No animal model reproduces MOGAD cortical encephalitis with spontaneous recurrent seizures; existing MOG-directed models largely reproduce white-matter experimental autoimmune encephalomyelitis. A useful model would require a conformationally appropriate human MOG antibody, permissive MOG-reactive T-cell inflammation, blood–CSF or subarachnoid access, a cytokine milieu resembling CCE, superficial cortical demyelination and prespecified electrographic spontaneous-seizure outcomes. Cortical pathology still rests on very few biopsies (49). The link to ictogenesis is therefore circumstantial, supported by epidemiological localization, subpial pathology, phenotype-associated CSF inflammation and independent evidence for downstream inflammatory ictogenesis (6, 8, 12, 13, 31, 49, 52, 55, 57).

The mechanisms described in Section 6 should therefore be read as a plausible pathway in which the upstream trigger is MOGAD-specific and most downstream effectors remain shared, extrapolated candidates. Distinguishing mechanism-level evidence from phenotype association and biological context is a principal research need.

Two additional considerations deserve mention. First, demyelination itself is not epileptogenically neutral: chronic cortical and callosal demyelination in experimental models is sufficient to produce spontaneous seizures, plausibly through loss of the myelin-dependent regulation of axonal excitability, altered conduction and secondary ion channel redistribution (58). Second, in MS — where cortical and temporal lobe damage is the strongest correlate of epilepsy — the association between gray matter pathology and seizures is well established (59), providing a demyelinating-disease precedent for the cortex-centric account advanced here.

## 6 Mechanisms of cortical hyperexcitability: established and inferred

How cortical inflammation in MOGAD produces seizures is not known. The account below is an inference: inflammatory signaling can lower seizure threshold, while seizures can amplify inflammation, with both innate and adaptive immune mechanisms implicated in epileptogenesis and human epilepsy (12, 13, 57, 60–62). **Table 2** uses defined evidence categories and deliberately avoids labeling the presence of a mediator or cell as direct mechanistic proof.

**Table 2:** Candidate pathways linking cortical neuroinflammation to hyperexcitability and the directness of supporting evidence.

Candidate mediator or pathway	Proposed effect on excitability	MOGAD-specific evidence	Evidence in epilepsy outside MOGAD
IL-1β/HMGB1–TLR4	NMDA-receptor potentiation and innate danger signaling	Extrapolated only: not tested in MOGAD tissue or CSF as an ictogenic mechanism	Mechanism-level experimental and human tissue evidence (63–66)
Tumor necrosis factor	AMPA/GABA-A receptor trafficking; context-dependent net effect	Extrapolated only	Mechanism-level but bidirectional evidence (67–69, 88)
IL-6	Context-dependent modulation of excitability, gliosis and barrier function	Phenotype association: higher CSF levels in combined ADEM/CCE than optic neuritis; no causal test (55)	Mixed, context-dependent experimental evidence (70, 71)
CXCL8/neutrophil-related signaling	Proposed protease/oxidant-mediated barrier injury	Phenotype association: CSF elevation in encephalitic phenotypes; correlational (52, 53, 55)	Contextual inflammatory evidence; no MOGAD-specific mechanism
CD68+ macrophages/microglia	Cytokine release and synaptic remodeling	Contextual cellular evidence: abundant CD68+ cells, without distinction between microglia and infiltrating macrophages or demonstration of ictogenic signaling (49)	Mechanism-level evidence in epilepsy models (69, 72)
Astrocyte glutamate transport	Reduced glutamate clearance	Not studied	Human and experimental epilepsy evidence (72–74)
Astrocyte Kir4.1/gap junctions	Impaired potassium buffering and spatial redistribution	Not studied	Human and experimental epilepsy evidence (75–79)
Complement	Hypothesized synaptic tagging and microglial elimination	Contextual, anatomically nonspecific and inconsistent: *in vitro* complement-dependent cytotoxicity and occasional tissue deposition; no cortical synaptic tagging (43–46, 49)	Mechanism-level experimental and human tissue evidence (80, 81)
Blood–brain/blood–CSF barrier	Antibody access; albumin–TGF-β astrocytic signaling	Clinical/radiological context supports barrier dysfunction, but the ictogenic albumin pathway is untested	Mechanism-level experimental evidence (82–87)

Evidence categories: mechanism-level, functional or causal evidence bearing on the proposed mechanism; phenotype association, an observational relationship with a seizure-prone MOGAD phenotype; contextual, presence of a relevant molecule, cell or anatomical condition without demonstration of the downstream mechanism; extrapolated only, evidence derived entirely from conditions outside MOGAD; not studied, no identified MOGAD-specific evidence.

Cytokines can alter synaptic transmission within minutes to hours. Interleukin-1β, acting through IL-1R1, activates Src-family kinases and enhances NMDA-receptor calcium influx (63). The parallel HMGB1/Toll-like receptor 4 axis is ictogenic in models and active in human epileptogenic cortex (64–66). Tumor necrosis factor can increase calcium-permeable AMPA-receptor surface expression and reduce GABA-A receptors, although receptor context can produce anticonvulsant effects (67–69, 88). IL-6 has context-dependent effects: experimental work supports increased excitability and gliosis in some settings, whereas IL-6 deficiency can increase seizure susceptibility in others (70, 71). In MOGAD, higher IL-6 in the combined ADEM/CCE group is a phenotype association, not a causal test (55). IL-6-directed treatment is therefore biologically motivated but not a proven antiseizure mechanism in MOGAD.

Glial mechanisms may sustain a state initiated by cytokines. Astrocytes clear synaptic glutamate through EAAT2/EAAT1 and buffer extracellular potassium through Kir4.1 and gap-junction coupling; these systems are altered in experimental and human epilepsy (72–79). Microglia produce tumor necrosis factor and participate in synaptic remodeling in epilepsy models. MOGAD lesions contain abundant CD68+ cells, but CD68 does not distinguish resident microglia from infiltrating macrophages and does not demonstrate ictogenic signaling (49).

Two additional pathways could connect humoral inflammation to cortical networks. Complement participates in synaptic elimination in epilepsy models (80, 81), but MOGAD evidence is limited to complement-fixing antibody function *in vitro* and occasional tissue deposition; cortical synaptic tagging has not been shown (43–46, 49). Barrier breakdown permits MOG-IgG access and is independently epileptogenic outside MOGAD: albumin–TGF-β signaling in astrocytes can alter potassium buffering and excitatory synaptogenesis (82–87). This pathway is a candidate explanation for persistent remodeling, not a demonstrated MOGAD mechanism. In MOG–NMDAR overlap, a direct synaptic mechanism is more secure because NMDAR antibodies impair inhibitory-neuron output (89).

This framework also offers a cautious interpretation of the clinical bimodality in Section 4.7. Most patients recover as inflammation resolves (8, 25, 26). In a minority, intense, prolonged or repeated attacks may permit durable remodeling; the associated clinical features include severe encephalitis, status epilepticus, recurrence and young age (6, 7, 19). Prompt seizure control may interrupt a reciprocal seizure–inflammation loop, while early immunotherapy addresses its cause (**Figure 2**). These propositions require prospective electroclinical testing rather than retrospective association.

**Figure 2:**
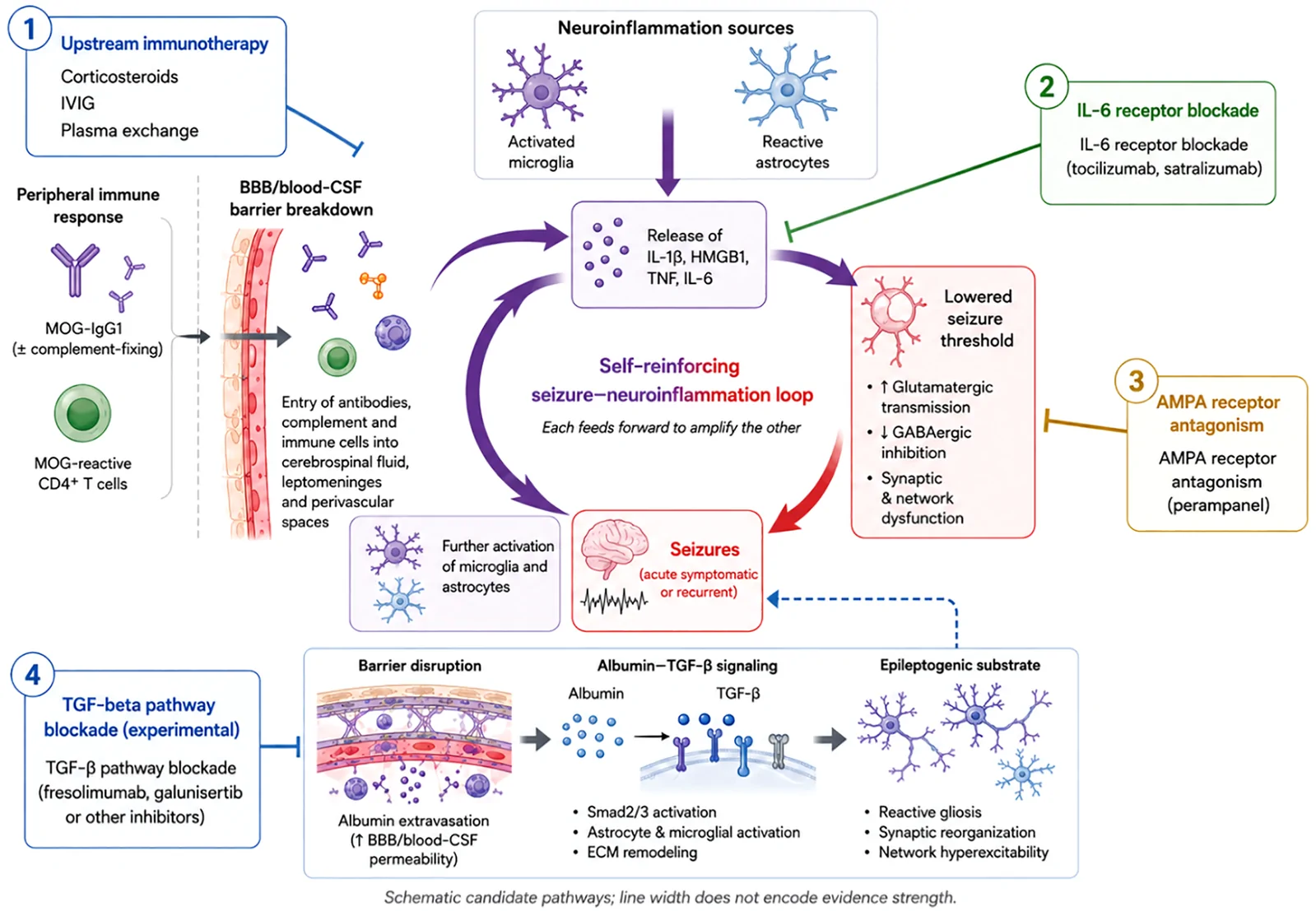
Simplified bidirectional seizure–neuroinflammation loop and candidate therapeutic interruption points. Immunotherapy targets the upstream MOGAD inflammatory attack; ASM provides symptomatic seizure control; selected cytokine-directed rescue treatment may be considered in refractory cases; TGF-β pathway blockade remains experimental. Arrow width does not encode evidence strength. The figure was created and revised using OpenAI image-generation tools and assembled by the authors. All schematic elements are original and no third-party copyrighted material was reproduced.

## 7 Treatment

### 7.1 Acute immunotherapy is the primary seizure treatment

The principal acute-treatment concept is to control both seizures and the underlying inflammation. Typical adult induction is intravenous methylprednisolone 1 g/day for 3–5 days. In children, consensus practice uses 20–30 mg/kg/day (maximum 1 g/day) for 3–5 days, followed by an individualized oral taper; in pediatric MOGAD, tapers extending over several weeks and sometimes up to approximately 3 months are used when relapse risk and toxicity permit (4, 5, 90, 91). Intravenous immunoglobulin or plasma exchange should be expedited in severe attacks or when meaningful improvement is absent after initial corticosteroids, rather than delayed through repeated ineffective courses. Evidence derives from consensus and observational cohorts, not randomized seizure-specific trials.

Taper duration should balance relapse prevention against corticosteroid toxicity. Earlier immunotherapy and longer courses are associated with lower pediatric relapse risk, and delayed steroid initiation was associated with later epilepsy diagnosis, but these observational data do not establish an optimal universal taper (19, 90).

### 7.2 The role, choice and duration of antiseizure medication

Because most MOGAD seizures are acute symptomatic, prolonged antiseizure medication (ASM) is often unnecessary once inflammation is controlled. There is no evidence-based fixed duration. A supervised taper can be considered after sustained seizure freedom, resolution of the acute lesion and inflammatory syndrome, and reassuring follow-up EEG when clinically indicated. Longer treatment is reasonable after recurrence outside attacks, status epilepticus, persistent epileptiform discharges, structural cortical sequelae or repeated encephalitic attacks (8, 11, 18).

ASM choice should follow seizure type, age, comorbidity and interaction profile. Levetiracetam, lacosamide and other broad-spectrum agents are commonly used. Perampanel has an experimental mechanistic rationale and observational experience in refractory status epilepticus, but no MOGAD-specific comparative evidence supports preferential use; it should not be selected solely to target a proposed inflammatory pathway (92–94).

### 7.3 Refractory and catastrophic presentations: the case for IL-6 blockade

Status epilepticus, super-refractory status epilepticus and NORSE require parallel critical-care seizure management and rapid evaluation for infectious, autoimmune and other causes (28, 29). International NORSE consensus recommends first-line immunotherapy within 72 hours in cryptogenic cases when a noninfectious cause is suspected and second-line immunotherapy within 7 days if the response is inadequate (95). IL-6 receptor blockade is a rational rescue option after exclusion of infection in selected refractory cases, supported by MOGAD cytokine associations and experience in cryptogenic NORSE or severe CNS inflammation, but it is not established as a routine MOGAD antiseizure treatment (28, 52, 55, 96–98). Other targeted immunomodulatory approaches, including JAK inhibition with tofacitinib, have been reported in refractory autoimmune encephalitis, although evidence remains limited and this strategy has not been established for MOGAD (99).

### 7.4 Maintenance immunotherapy

Relapse risk is substantial — 50% in one adult CCE series (25), 60% in a seizure cohort (8) — and determines the need for maintenance therapy. Options with the best supporting evidence in MOGAD are maintenance intravenous immunoglobulin, which is associated with relapse prevention in adults (100), and, increasingly, IL-6 receptor blockade: a recent multicenter analysis supports IL-6 receptor blockade for relapse prevention in MOGAD (101), and comparative work has begun to define its place relative to other agents in MOGAD and NMOSD (102). Treatment response in children with relapsing MOG antibody disease has also been characterized and supports individualized escalation (103). Persistently high MOG-IgG titers and specific serological trajectories carry prognostic information for relapse and may help select patients for maintenance therapy (104, 105).

From the seizure perspective, the relevant point is that relapse prevention is also, indirectly, epilepsy prevention: recurrent encephalitic attacks are among the strongest predictors of chronic epilepsy (7, 19). Seizure freedom does not equal full recovery. Children may have residual attention, memory, behavior, fatigue or school-participation difficulties after encephalitis or ADEM; in one pediatric cohort, 39.5% required academic or educational intervention (106). Follow-up should therefore include age-appropriate developmental, behavioral and neuropsychological screening, school feedback and referral for formal assessment or rehabilitation when concerns persist.

## 8 Conclusion

Seizures are an age-dependent and mechanistically informative feature of MOGAD. The available evidence supports an interaction among demyelination, lesion anatomy and inflammatory composition: a CD4+ T-cell- and macrophage-rich process centered on leptomeninges and superficial cortex can engage shared pathways of inflammatory ictogenesis. However, most downstream pathways remain extrapolated, IL-6 and CXCL8 data are associative, and sparse complement deposition cautions against a simple antibody–complement account. In most patients seizures remit with control of inflammation; a minority—disproportionately children—develop recurrent seizures or chronic epilepsy, and some survivors have cognitive, behavioral or academic sequelae despite seizure freedom.

Clinically, MOG-IgG testing should be phenotype-directed, peri-ictal MRI change and mimics should be considered, and acute management should combine seizure control with prompt immunotherapy (summarized in **Table 3**). Scientifically, this review advances two falsifiable predictions: lesion location should modify seizure risk independently of overall inflammatory burden, and persistent barrier or CSF inflammatory abnormalities should identify patients at risk for chronic epilepsy. Testing these predictions requires prospective cohorts with standardized EEG, serial MRI, compartment-resolved immune measures and neuropsychological outcomes, together with a cortical MOGAD model that includes spontaneous seizures.

**Table 3:** Practical evaluation and management of MOGAD-associated seizures.

Domain	Practical approach	Pediatric considerations	Evidence limits
Testing and assay interpretation	Test serum MOG-IgG when seizures accompany a compatible MOGAD core phenotype or inflammatory cortical syndrome; prefer a validated live CBA; interpret low-positive or fixed-CBA results with supporting features and exclusion of mimics	Avoid indiscriminate testing of isolated uncomplicated seizures	Positive predictive value falls at low titer and low pretest probability (3, 36, 37)
MRI, CSF and differential diagnosis	Use contrast MRI, diffusion imaging and CSF; distinguish HSV, MELAS, Rasmussen encephalitis, neuronal-surface antibody encephalitis and peri-ictal change; repeat MRI when initial imaging is normal but suspicion persists	Consider opening pressure and developmental/metabolic mimics	Peri-ictal and inflammatory changes can coexist
EEG	Obtain routine EEG; use continuous EEG for persistent altered consciousness, suspected nonconvulsive seizures or status epilepticus	Lower threshold for continuous monitoring in encephalopathy	A normal routine EEG does not exclude CCE
Acute immunotherapy	Adults: intravenous methylprednisolone 1 g/day for 3–5 days, then individualized taper	20–30 mg/kg/day (maximum 1 g/day) for 3–5 days; taper over weeks and sometimes up to ~3 months when justified	Consensus and observational evidence; balance relapse prevention against toxicity (4, 5, 90, 91)
Escalation	Expedite IVIG or plasma exchange for severe disease or inadequate meaningful response after initial corticosteroids	Consider age, hemodynamic access and intensive-care context	No seizure-specific randomized threshold
NORSE/refractory status	Parallel critical-care treatment and etiological workup; first-line immunotherapy within 72 h and second-line by 7 days when noninfectious NORSE remains likely	Coordinate pediatric neurocritical care and immunology	Consensus-based; targeted cytokine therapy remains selected rescue treatment (95)
ASM duration	No fixed duration; consider supervised taper after sustained seizure freedom, inflammatory and MRI resolution, and reassuring EEG when indicated	Review cognition, behavior and school effects of medication	Continue longer after recurrence outside attacks, status epilepticus, epileptiform EEG or structural sequelae
Follow-up	Monitor relapse, seizures, MRI and treatment toxicity	Add developmental, behavioral, neuropsychological and academic screening	Seizure freedom does not establish full functional recovery (106)
